# Dynamic Landscapes of tRNA Transcriptomes and Translatomes in Diverse Mouse Tissues

**DOI:** 10.1016/j.gpb.2022.07.006

**Published:** 2022-08-08

**Authors:** Peng Yu, Siting Zhou, Yan Gao, Yu Liang, Wenbing Guo, Dan Ohtan Wang, Shuaiwen Ding, Shuibin Lin, Jinkai Wang, Yixian Cun

**Affiliations:** 1 Center for Translational Medicine, Precision Medicine Institute, The First Affiliated Hospital, Sun Yat-sen University, Guangzhou 510080, China; 2 Department of Radiation Oncology, Affiliated Cancer Hospital & Institute of Guangzhou Medical University, Guangzhou 510080, China; 3 Department of Medical Informatics, Zhongshan School of Medicine, Sun Yat-sen University, Guangzhou 510080, China; 4 Center for Stem Cell Biology and Tissue Engineering, MOE Key Laboratory for Stem Cells and Tissue Engineering, Sun Yat-sen University, Guangzhou 510080, China; 5 RIKEN Center for Biosystems Dynamics Research, Kobe 650-0047, Japan; 6 Graduate School of Biostudies, Kyoto University, Kyoto 606-8501, Japan; 7 Wuya College of Innovation, Shenyang Pharmaceutical University, Shenyang 110016, China; 8 RNA Biomedical Institute, Sun Yat-sen Memorial Hospital, Sun Yat-sen University, Guangzhou 510080, China

**Keywords:** Translational efficiency, Tissue-specific, tRNA expression, Codon usage bias, Amino acid composition

## Abstract

Although the function of tRNAs in the translational process is well established, it remains controversial whether tRNA abundance is tightly associated with **translational efficiency** (TE) in mammals. Moreover, how critically the expression of tRNAs contributes to the establishment of **tissue-specific** proteomes in mammals has not been well addressed. Here, we measured both **tRNA expression** using demethylase-tRNA sequencing (DM-tRNA-seq) and TE of mRNAs using ribosome-tagging sequencing (RiboTag-seq) in the brain, heart, and testis of mice. Remarkable variation in the expression of tRNA isodecoders was observed among different tissues. When the statistical effect of isodecoder-grouping on reducing variations is considered through permutating the anticodons, we observed an expected reduction in the variation of anticodon expression across all samples, an unexpected smaller variation of anticodon usage bias, and an unexpected larger variation of tRNA isotype expression at amino acid level. Regardless of whether or not they share the same anticodons, the isodecoders encoding the same amino acids are co-expressed across different tissues. Based on the expression of tRNAs and the TE of mRNAs, we find that the tRNA adaptation index (tAI) and TE are significantly correlated in the same tissues but not between tissues; and tRNA expression and the **amino acid composition** of translating peptides are positively correlated in the same tissues but not between tissues. We therefore hypothesize that the tissue-specific expression of tRNAs might be due to post-transcriptional mechanisms. This study provides a resource for tRNA and translation studies, as well as novel insights into the dynamics of tRNAs and their roles in translational regulation.

## Introduction

The genetic information is transmitted from DNA to RNA, and then to proteins. However, the correlation between mRNA abundance and protein expression level is far from linear, suggesting that the translation process plays an indispensable role in determining the output of proteins [1]. During protein synthesis, tRNAs decode the mRNA templates via codon–anticodon pairing and deliver the amino acids to the corresponding polypeptide chain in the ribosomes [2]. tRNAs are small non-coding RNAs, 70–90 nt in length, transcribed by RNA polymerase III (RNAPIII), and constitute 4%–10% of the total RNA in a cell [3]. Although there are only 20 amino acids and 64 codons, about 400 nuclear-derived tRNAs have been annotated in mammals (*e.g.*, 429 and 401 annotated tRNA genes in human and mouse genomes, respectively) in addition to 22 mitochondrial tRNAs (mt-tRNAs) [4]. tRNA transcripts that carry the same anticodons but different sequences are termed isodecoders [5], while different tRNA species accepting the same amino acids are termed isoacceptors [3]. There are 49 and 47 isoacceptors annotated in human and mouse genomes, respectively [6], [7].

In bacteria and yeast, the tRNA abundance correlates well with the codon usage of highly translated genes (HTGs) [8], [9], [10]. In mammals, the relationship between them is still in debate. Several studies have reported correlations between the tRNA abundance and codon usage. For example, Dittmar et al*.* reported that the tRNA abundance is significantly correlated with the codon usage of tissue-specific and highly expressed genes [11]. Gingold et al*.* found that tRNAs induced in proliferative cells or differentiated cells often decode codons enriched in mRNAs related to cell autonomy and multicellularity [12]. Waldman et al*.* reported better adaptation of tissue-specific genes with the tRNA pool when compared with non-specific genes [13]. Hernandez-Alias et al*.* reported that the tissue-specific tRNA pools determine the translational efficiency (TE) of proliferation-related genes [14]. Najafabadi et al*.* reported that the codon usage correlates with the TE of genes involved in adaptation to environmental and physiological changes [15]. However, other studies have reported that the correlations are poor. Sémon et al*.* reported that the significant differences in synonymous codon usages between tissues are not due to translational selection [16]. Kanaya et al*.* reported that the ribosome genes and histone genes show no difference in codon usage, implying no translational regulation through tRNAs [17]. Thus, it is unclear how tRNA expression profiles are correlated to the TE of specific transcripts.

To address this issue, quantitative tRNA expression evaluation is desirable. However, due to the stable structure and diverse post-transcriptional modifications of tRNAs which interfere with reverse transcription efficiency and adaptor ligation, it has been difficult for standard sequencing methods to detect tRNA pools efficiently and quantitatively. Most studies have utilized microarrays or RNAPIII chromatin immunoprecipitation followed by sequencing (ChIP-seq) to identify tRNA transcriptomes. In recent years, more next-generation sequencing methods have been developed to measure the abundance of tRNAs [18], [19], [20], [21], [22]. However, none of these studies have compared the tRNA abundance with matched translatome data. Therefore, whether the dynamics of tRNA expression contribute to the establishment of tissue-specific translatomes in mammals has not been well addressed.

Although still largely elusive, the regulation of tRNA expression can be possibly mediated by transcriptional and post-transcriptional mechanisms. On the one hand, the occupancy of RNAPIII as considered at the isoacceptor family level was invariant in multiple mammalian tissues [7]. On the other hand, the RNA modification and structure of tRNAs can regulate the ribonuclease-catalyzed degradation of tRNAs [23], [24], [25]. It was also reported that multiple tRNAs were degraded when histidine or leucine becomes limited, suggesting that the tRNA expression was also under post-transcriptional regulation [26].

In this study, to overcome the difficulty of measuring tRNA expression, we applied the demethylase-tRNA sequencing (DM-tRNA-seq) method developed by Zheng et al. [27] to evaluate the diversity of tRNA pools in three mouse tissues (brain, heart, and testis). The DM-tRNA-seq utilizes engineered demethylase AlkB to remove base methylation on tRNAs and can measure the tRNA transcriptome efficiently and quantitatively [27]. Meanwhile, we applied ribosome-tagging sequencing (RiboTag-seq) to capture the ribosome-associated mRNAs in the same mouse tissues [28]. We found various degrees of variations of tRNA expression at the isodecoder, isoacceptor, and amino acid isotype levels among different mouse tissues, suggesting the dynamic expression of tRNAs. We then found that the tRNA adaptation index (tAI) was significantly correlated with TE intra- but not inter-tissues. Our study suggests that the differential tRNA expression between tissues is not likely to contribute to tissue-specific translatomes, but may result from post-transcriptional regulation of tRNAs.

## Results

### Dynamic expression of tRNA isodecoders among different mouse tissues

**Figure 1:**
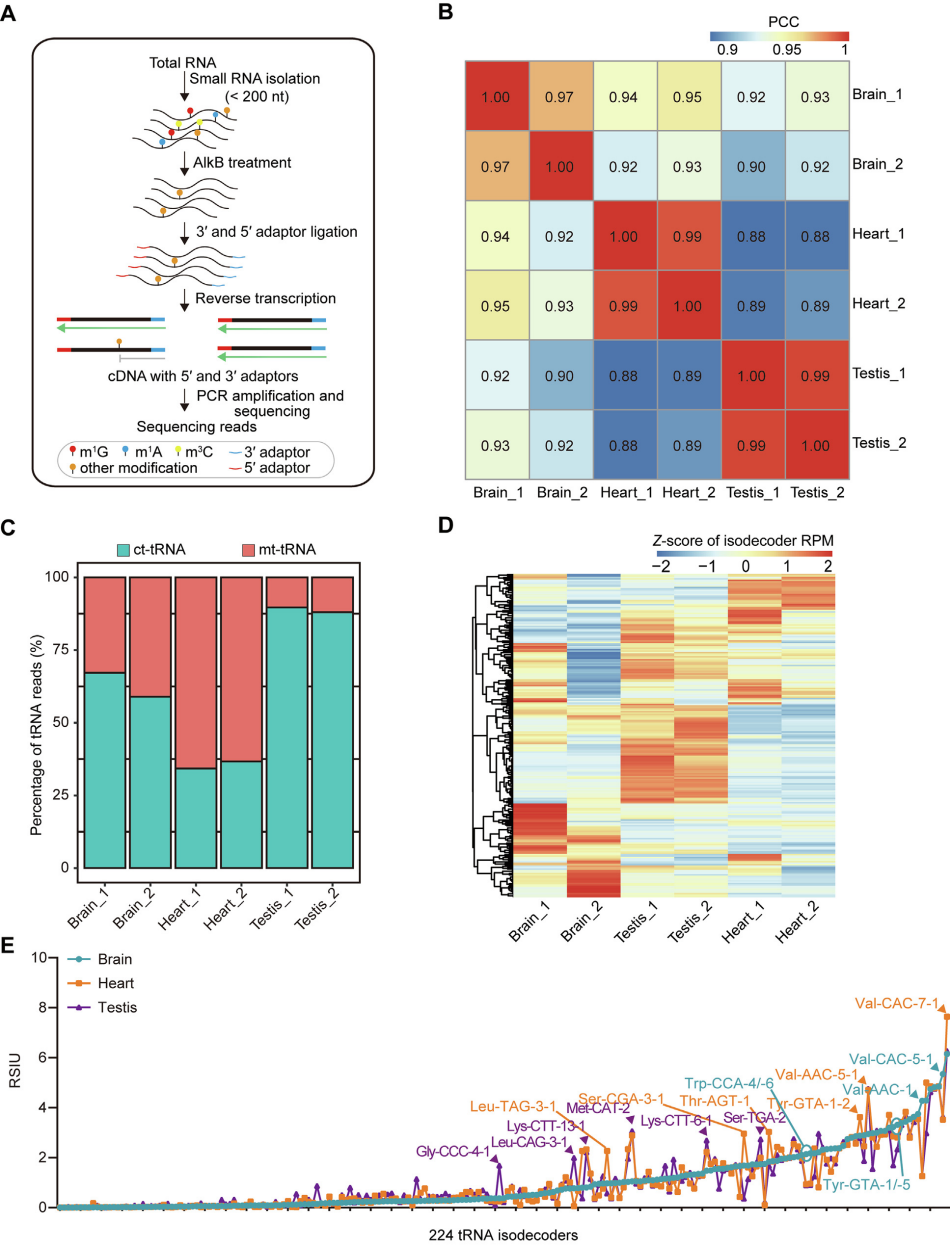
**Dynamic expression of tRNA isodecoders among different mouse tissues** **A.** Schematic representation of DM-tRNA-seq based on AlkB demethylation. **B.** Heatmap of pairwise PCCs of tRNA isodecoder expression among the six samples of the three mouse tissues. **C.** Stacked bar plot depicting the percentages of ct-tRNA reads and mt-tRNA reads in three mouse tissues. **D.** Heatmap showing the expression of tRNA isodecoders (*Z*-score) in the six samples of the three mouse tissues. **E.** Line chart comparing the strength of isodecoders’ usage bias in different tissues as measured by RSIU. The representative isodecoders with high tissue specificity are indicated. PCR, polymerase chain reaction; PCC, Pearson correlation coefficient; ct-tRNA, cytosolic tRNA; mt-tRNA, mitochondrial tRNA; RPM, reads per million mapped reads; RSIU, relative synonymous isodecoder usage.

**Supplementary Figure S1:**
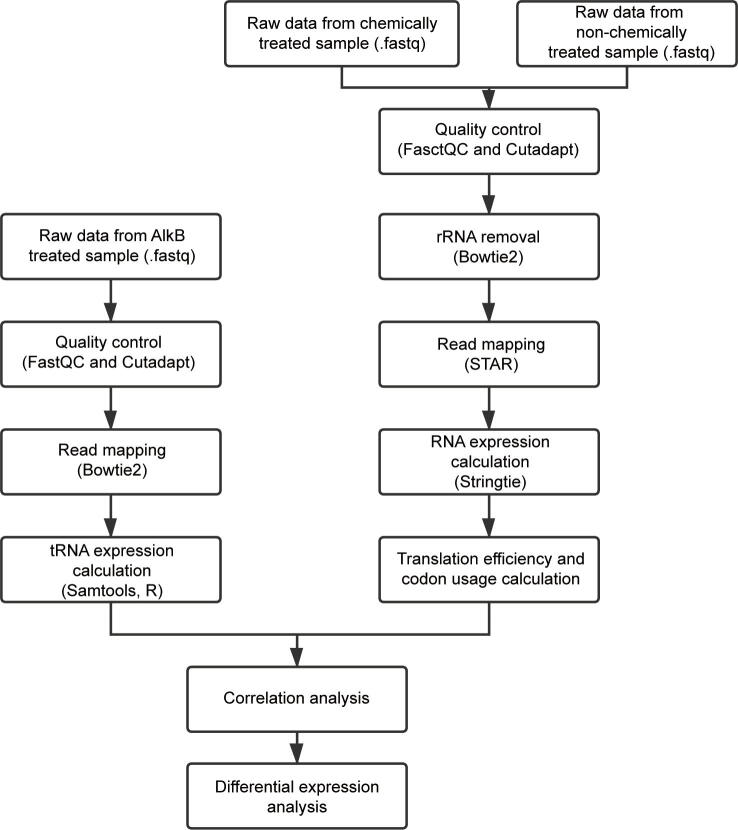
**The bioinformatics analysis flow chart** The left flow chart shows the bioinformatic analysis steps of DM-tRNA-seq data; and the right flow chart shows the bioinformatic analysis steps of RiboTag-seq data.

In order to systematically elucidate the tissue specificity of tRNA expression, we obtained total RNA samples from three tissues (brain, heart, and testis) of adult male *CMV-Cre:RiboTag* mice, and generated tRNA libraries for DM-tRNA-seq with two biological replicates (Figure 1A). The reads per million mapped reads (RPM) was calculated for each tRNA annotated in genomic tRNA database GtRNAdb [4] and mt-tRNA database mitotRNAdb [29] (Figure S1; Table S1). As shown in Figure 1B, the biological replicates of the same tissues were highly similar to each other and clustered together, suggesting tissue-specific expression of tRNAs. We noted that the tRNA expression pattern of the brain tissue is less reproducible than that of the heart and testis, possibly reflecting the higher cell heterogeneity of the brain. In addition, we found that mt-tRNAs accounted for 11.1% of the total detected tRNAs in the testis but 64.4% in the heart and 38.9% in the brain, which is consistent with the orders of energy demands in these tissues (Figure 1C). Since the dynamics of mt-tRNA contents are more likely to reflect the dynamics of the number of mitochondria in the cells, we focused on the dynamics of cytosolic tRNAs (ct-tRNAs), which may relate to the translational regulation of nuclear-derived genes.

Differential expression analysis of tRNA isodecoders was performed on three tissues using DESeq2 [30]. Among the 224 detected tRNA isodecoders with unique sequences, 131 (58%) of them had significantly differential expression [false discovery rate (FDR) < 0.05] across the three tissues (Figure 1D). To further elucidate the potential role of expression regulation of tRNA isodecoders on translation, we defined a metric, relative synonymous isodecoder usage (RSIU), to analyze the usage bias of synonymous isodecoders with the same anticodons (details in Materials and methods). As shown in Figure 1E, we observed remarkable differences in the RSIU values among the tissues, and 36 of the 224 isodecoders showed more than 2-fold higher RSIU values in one tissue than those in the other two tissues. For example, the RSIU value of isodecoder Gly-CCC-4-1 in the testis was 6.9 and 4.5 folds of that in the heart and brain, respectively.

### Tissue-specific expression of isodecoders results in tissue-specific expression rather than tissue-specific usage bias of anticodons

**Figure 2:**
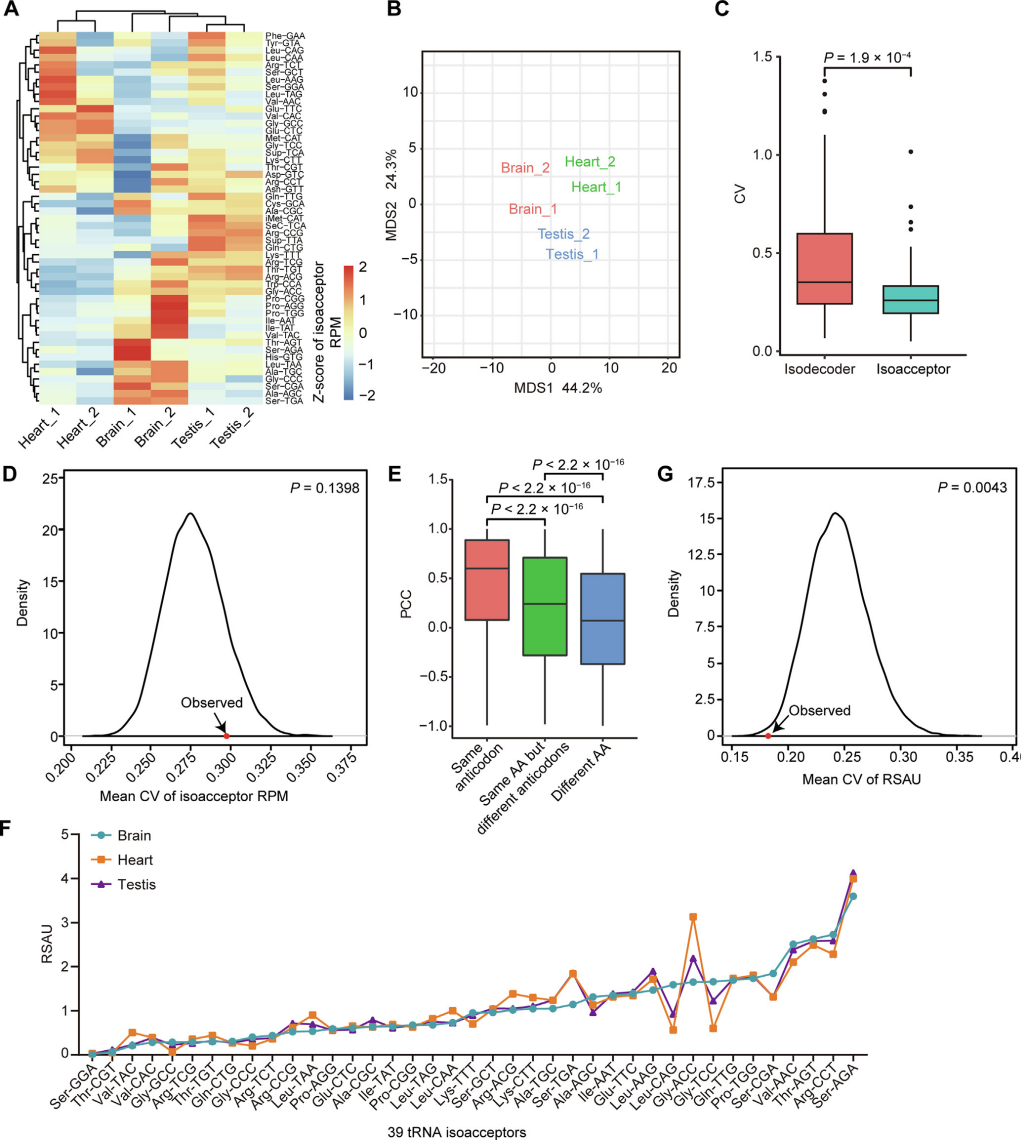
**Tissue-specific expression of isodecoders resultsin tissue-specific expressionrather than tissue-specificusage bias of anticodons** **A.** Heatmap showing the *Z*-scores of tRNA isoacceptor expression in the six samples of the three mouse tissues. **B.** MDS plot displaying the clustering of the six samples of the three mouse tissues according to the tRNA expression profiles. **C.** Box plot comparing the CVs of isodecoders and isoacceptors among the six samples of the three mouse tissues. *P* value was calculated by two-tailed Wilcoxon test. **D.** Density plot showing the distribution of mean CVs of isoacceptor expression across the six samples for 10,000 permutations as well as the observed as indicated by red dot and arrow. *P* value was calculated as the proportion of permutations with greater X-axis values than the observed. **E.** Box plot comparing the pairwise PCCs of three groups of isodecoders: same anticodon, same AA but different anticodons, and different AA, according to the corresponding anticodons and amino acids of the pairs of two isodecoders. *P* values were calculated by two-tailed Wilcoxon test. **F.** Line chart comparing the strength of anticodon usage bias based on DM-tRNA-seq in three tissues as measured by RSAU. **G.** Density plot showing the distribution of mean CVs of RSAU values across the six samples for 10,000 permutations as well as the observed as indicated by red dot and arrow. *P* value was calculated as the proportion of permutations with smaller X-axis values than the observed. MDS, multidimensional scaling; CV, coefficient of variation; AA, amino acid; RSAU, relative synonymous anticodon usage.

**Supplementary Figure S2:**
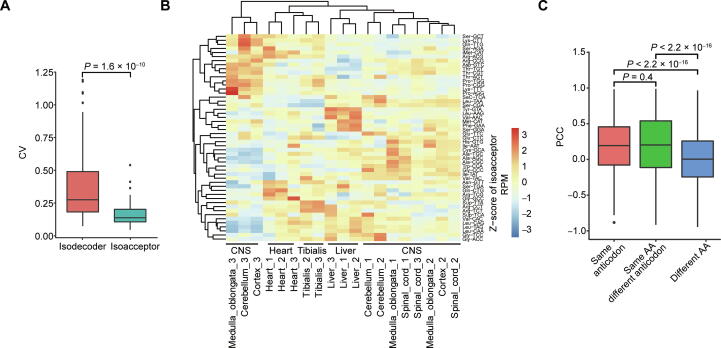
**Dynamic expression of anticodons using published QuantM-tRNA seq data A.** Comparison of the CVs of isodecoders and isoacceptors among seven tissues. *P* values of two-tailed Wilcoxon tests are indicated. **B.** Heatmap representing the Z-scores of tRNA reads which collapsed by known isoacceptor groups in seven tissues. Two outliers were removed from the analysis (Cortex_1 and Tibialis_1). **C.** Box plot comparing the pairwise PCCs of three groups of isodecoders: “same anticodon”, “same amino acid but different anticodons”, and “different amino acids” according to the corresponding anticodons and amino acids of the pairs of two isodecoders.

To assess tRNA expression at the anticodon level, the 224 ct-tRNAs identified by DM-tRNA-seq were separated into 51 groups, including 47 isoacceptors with unique anticodons as well as 4 special tRNA groups (tRNA-SeC-TCA, tRNA-Sup-TTA, tRNA-Sup-TCA, and tRNA-iMet-CAT). The expression heatmap of these 51 tRNA groups demonstrated the differential expression across the three tissues (Figure 2A). We found that the samples of the same tissues were clustered together according to the expression of the 51 tRNA groups, suggesting tissue-specific expression of anticodons (Figure 2A and B). Of note, we realized that the coefficient of variations (CVs) of the expression of the 47 isoacceptors among tissues were significantly smaller than those of isodecoders (Figure 2C), consistent with the recent study reporting a milder difference in isoacceptor expression among tissues [21]. To validate our results, we also compared the CVs of isoacceptors and isodecoders using one published tRNA dataset examined by a different technology QuantM-tRNA seq [21]. Similarly, we observed greatly reduced CVs of the expression of isoacceptors than isodecoders (Figure S2A). To test whether the relatively small variations of isoacceptor expression were due to genuinely tissue-specific expression of anticodons, we calculated the expression of isoacceptors using QuantM-tRNA seq data. Based on the heatmap of *Z*-score transformed expression of isoacceptors, we found reproducible tissue-specific isoacceptor expression, although different regions of brain were not largely distinct from each other (Figure S2B), which is consistent with our results using DM-tRNA-seq, suggesting the tissue-specific expression of isoacceptors.

Then we asked why the CVs of isoacceptors were smaller than isodecoders. We suspected that averaging the subgroups of isodecoders would reduce variations due to statistical principles. We therefore asked whether the reduced variations of isoacceptors among tissues were simply statistically due to the random combinations of isodecoders. For this purpose, we performed permutation analyses by randomly permutating anticodon of the isodecoders and regrouped them into isoacceptors according to the permutated anticodon. We found that the observed mean CV of the isoacceptors among tissues was greater than 86% of 10,000 permutations, indicating a non-significant difference (Figure 2D). The results suggest that the dynamic expression of isoacceptors is simply a reflection of the dynamic expression of isodecoders. In other words, although not so remarkable, the dynamic expression of isoacceptors is genuine.

We then turned to uncover the relationships among the expression levels of the isodecoders. We calculated the Pearson correlation coefficient (PCC) of any two isodecoders across all six samples (Figure 2E). The PCCs of isodecoder pairs encoding different amino acids, which are the most unrelated isodecoders, were around 0, suggesting that the unrelated isodecoders are independently regulated. Interestingly, we found that the PCCs between the isodecoder pairs with different anticodons but encoding the same amino acids were significantly greater than those of isodecoder pairs encoding different amino acids. In addition, the isodecoder pairs with the same anticodons turned out to have the highest PCCs. To confirm, we performed the same analyses using the published dataset of tRNA expression in multiple mouse tissues based on a different tRNA sequencing technology QuantM-tRNA seq [21]. We observed similar results that the isodecoder pairs with the same anticodons and the pairs with different anticodons but encoding the same amino acids were almost equal and both had significantly greater PCCs than the unrelated pairs (Figure S2C). These results suggest that functionally related isoacceptors do not randomly fluctuate among different tissues but are associated and possibly co-regulated across different tissues, especially at the amino acid level.

Nevertheless, it is an interesting question whether the tissue-specific expression of isoacceptors results in tissue-specific usage bias of tRNA anticodons encoding the same amino acids, which would subsequently lead to differential TEs in different tissues. To address this question, we calculated the relative synonymous anticodon usage (RSAU) values according to the expression of anticodons in the three tissues, respectively (details in Materials and methods). We found that the overall variations of RSAU values across tissues were much lower than RSIU values (Figures 1E and 2F). We further found that the CVs of RSAU values among three tissues were significantly smaller than random permutations (Figure 2G). The mean CVs of permutated RSAU values greater than the mean of observed CV could be obtained 9957 times out of 10,000 permutations, suggesting that the variations of RSAU values among different tissues are prohibited. The aforementioned results demonstrate that the distinctive expression of tRNA isoacceptors does not play a vital role in selecting specific synonymous anticodons or determining the TEs in different tissues.

**Figure 3:**
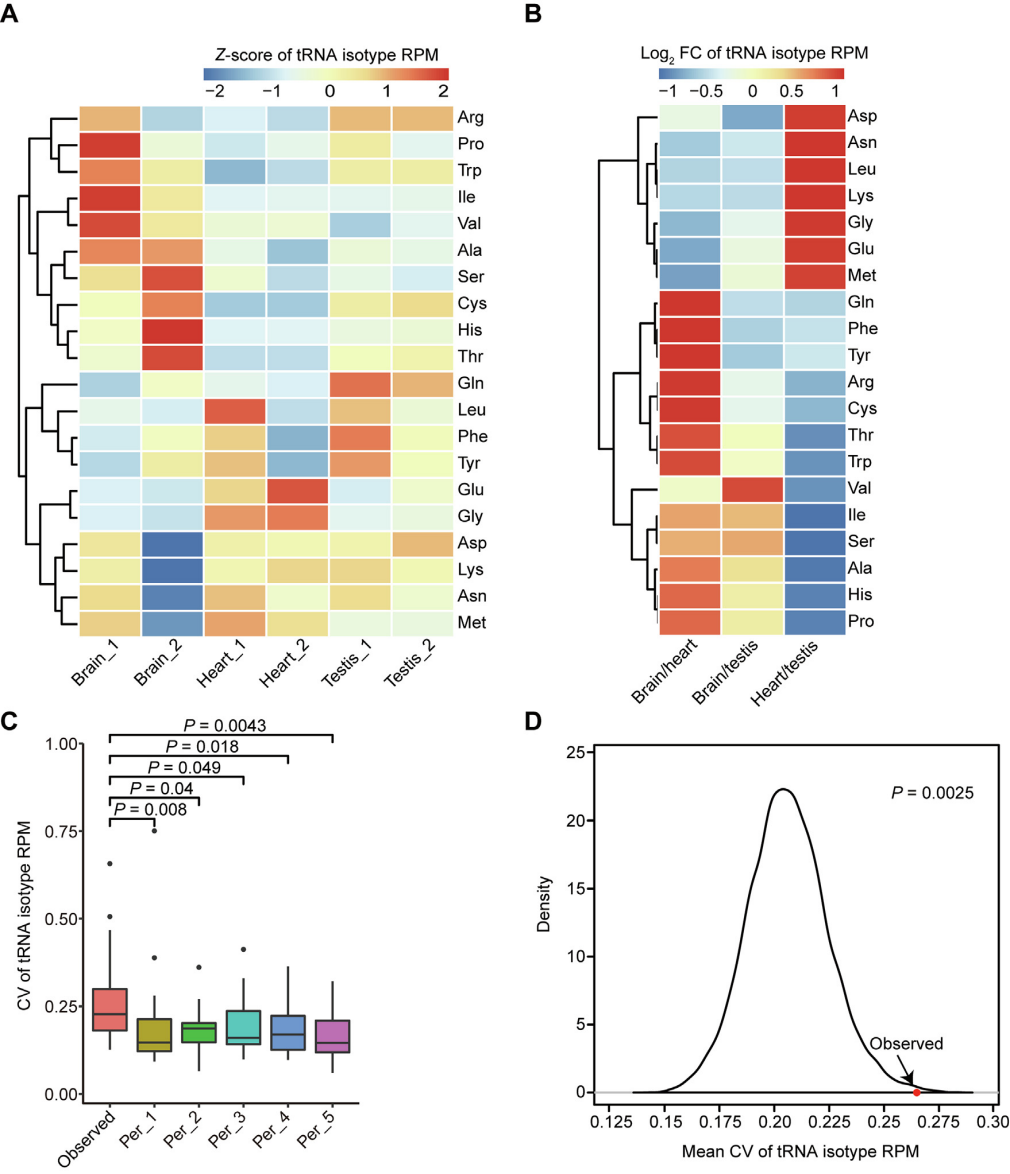
**Tissue**-**specific tRNA isotype expression at amino acid level** **A.** Heatmap showing the *Z*-scores of tRNA isotype expression in the six samples of three mouse tissues. **B.** Heatmap showing the log_2_-transformed FCs of tRNA isotype expression for the pairwise comparisons of the three tissues. **C.** Box plot comparing the CVs of tRNA isotype expression across six samples (observed) with five random permutations. *P* values were calculated by two-tailed Wilcoxon test. **D.** Density plot showing the distribution of mean CVs of tRNA isotype expression across the six samples for 10,000 permutations as well as the observed as indicated by red dot and arrow. *P* value was calculated as the proportion of permutations with greater X-axis values than the observed. FC, fold change.

Because the isodecoders encoding the same amino acids tend to be co-regulated, we speculated that the diversity of tRNA pools is most likely to match the amino acid composition within specific physiological states during the translation process. We then tested whether the tRNA isotype expression at amino acid level also had tissue specificity by combing the tRNAs encoding the same amino acids. As shown in Figure 3A and B, there is obvious tissue-specific tRNA isotype expression. In addition, the mean CV of isotype expression across these three tissues is greater than all 10,000 permutations, suggesting that there is genuine tissue-specific isotype expression (Figure 3C and D). These results together with the aforementioned results imply that the dynamic regulation of tRNAs among tissues is more likely a reflection of tissue-specific needs of tRNAs encoding specific amino acids rather than optimizing the codon usages for efficient translation.

### RiboTag-seq analysis of translatomes in multiple mouse tissues

**Figure 4:**
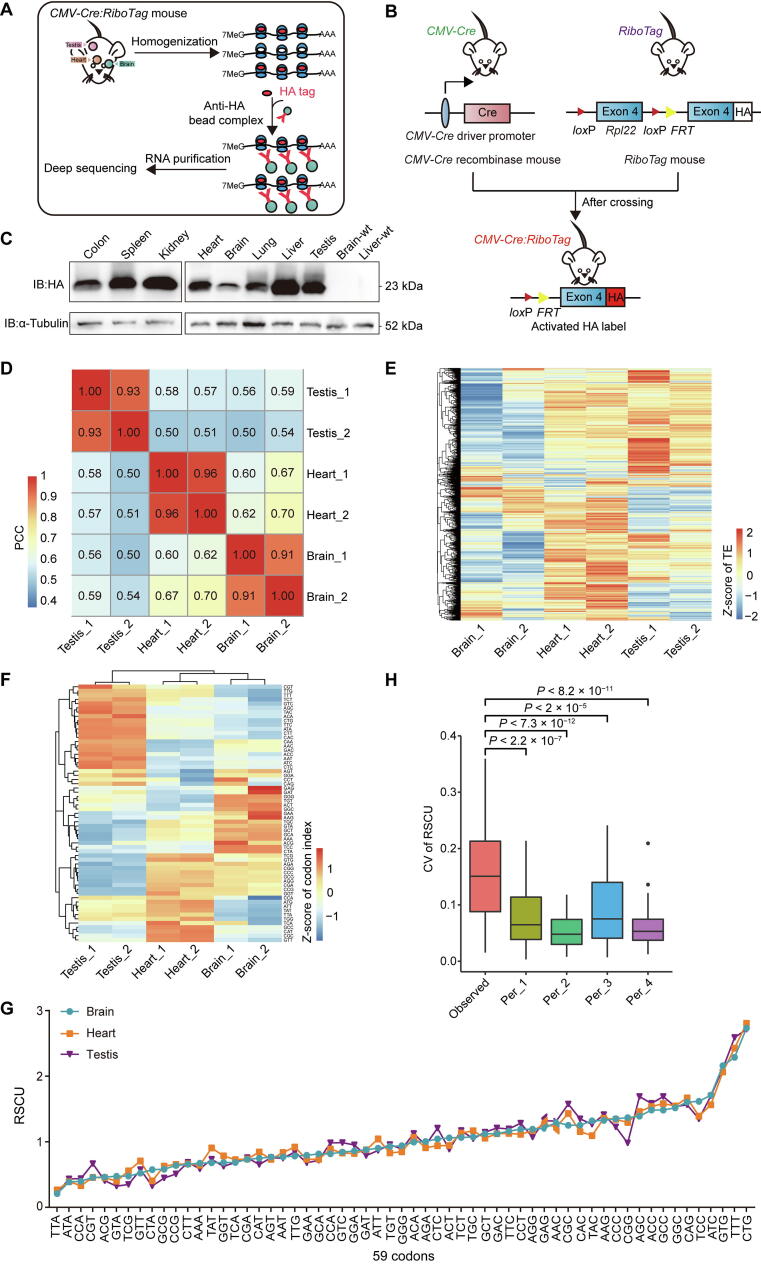
**RiboTag-seq analysis of translatomes in multiple mouse tissues** **A.** Overview of RiboTag-seq technology. **B.** Diagram depicting the *RiboTag* mouse systems. **C.** Western blot analysis of RPL22-HA in different tissues of *CMV-Cre:RiboTag* mouse. **D.** Heatmap showing pairwise PCCs among the six mouse samples in three tissues based on the FPKMs of genes in input samples of RiboTag-seq. **E.** Heatmap showing the *Z*-scores of TEs in six samples of three mouse tissues. **F.** Heatmap showing the *Z*-scores of codon indexes of top 5% HTGs in six samples of three mouse tissues. **G.** Line chart comparing the strength of codon usage bias in different tissues as measured by RSCU. **H**. Box plot comparing the CVs of RSCU values of the top 5% HTGs (observed) across six samples with four random permutations. *P* values were calculated by two-tailed Wilcoxon test. HA, hemagglutinin; 7MeG, *N*^7^-methylated guanosine; wt, wild-type; FPKM, fragments per kilobase per million mapped of fragments; TE, translational efficiency; HTG, highly translated gene; RSCU, relative synonymous codon usage.

**Supplementary Figure S3:**
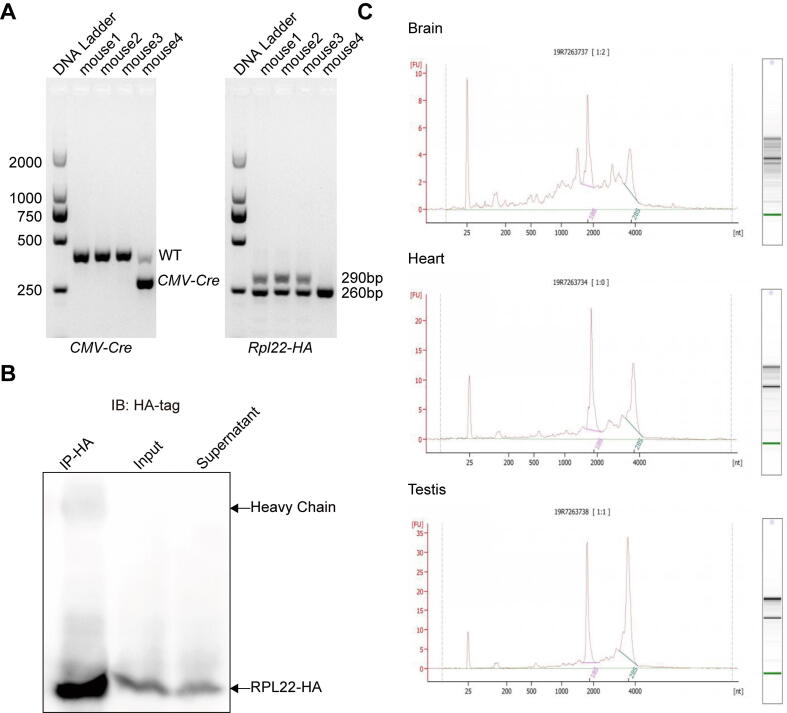
**RiboTag-seq analysis of translatomes in multiple mouse tissues reveals tissue-specific translational efficiency and codon usage biases A.** PCR products using primers that amplify CMV-Cre recombinase and the loxP-containing intron sequence of the *Rpl22* gene. The PCR product of *Rpl22* gene activated by CMV-Cre recombinase is 260 bp, and the non-activated PCR product is 290 bp. **B.** Western blots using an anti-HA antibody demonstrate the presence of RPL22-HA specifically in anti-HA pellets versus supernatant. **C.** Agilent Technologies 2100 Bioanalyzer (Catalog No. G2939BA, Agilent Technologies, Palo Alto, Calif.) electropherogram analysis of total RNAs from brain, heart, and testis immunoprecipitates. WT, wild type; IP, immunoprecipitation.

To further elucidate whether dynamic tRNA expression contributes to the establishment of tissue-specific translatomes, we performed RiboTag-seq in the same samples that were applied to DM-tRNA-seq. The RiboTag-seq technology takes advantage of RPL22, a component of the 60S subunit of ribosome, to capture the actively translating ribosomes (Figure 4A). The expression of RPL22-HA protein can be activated by Cre recombinase-mediated replacement of exon 4 with an HA-tagged exon 4 of *Rpl22* [31]. To create a line of mice constitutively expressing RPL22-HA protein in multiple tissues, *RiboTag* mice were mated with *CMV-Cre* mice (Figure 4B). We validated the heterozygous *CMV-Cre* and homozygous *Rpl22-HA* alleles in the genomes of offspring, and confirmed the expression of RPL22-HA protein in homogenate of multiple tissues, followed by efficient immunoprecipitating (Figure 4C, Figure S3A–C).

**Supplementary Figure S4:**
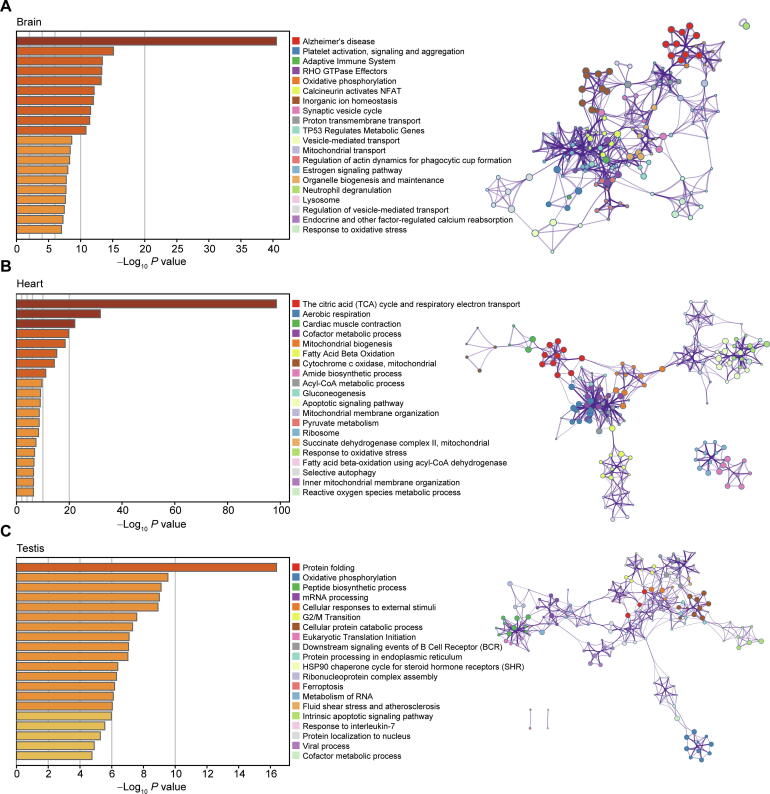
**Metascape enrichment analysis of HTGs in different tissues A.** Metascape enrichment analysis of top 5% HTGs in brain. **B.** Metascape enrichment analysis of top 5% HTGs in heart. **C.** Metascape enrichment analysis of top 5% HTGs in testis.

Since RiboTag-seq only sequenced the RNAs bound by the translation factor RPL22, we calculated the translation levels, which were represented by the gene expression levels of immunoprecipitation RNAs (IP), as well as TEs, which were the translation levels normalized by the expression of input RNAs (Figure S1; Table S2). Strong tissue-specific gene expression as well as TEs were observed (Figure 4D and E). Gene Ontology (GO) analysis and Kyoto Encyclopedia of Genes and Genomes (KEGG) analysis of the HTGs (top 5%) revealed enrichment for tissue development or tissue physiology-related processes and pathways (Figure S4A–C). We then asked whether the composition of codons was different among these tissues. For this purpose, we defined a metric of codon index (details in Materials and methods), which is the proportion of specific codons in all of the top 5% HTGs weighted by the translation level of each gene. As shown in Figure 4F, there are distinctive codon indexes among the three tissues, suggesting that it might be necessary for tissue-specific tRNA pools. To test whether the usage biases of synonymous codons also differ among tissues, for each codon, we modified the previously defined metric relative synonymous codon usage (RSCU) [32] by adding IP fragments per kilobase per million mapped of fragments (FPKM) as the weight of each gene, which is the observed IP FPKM weighted frequency of specific codon divided by the frequency expected under the assumption of equal usage of the synonymous codons. Based on the top 5% HTGs of each tissue, we found moderate differences among the brain, heart, and testis (Figure 4G). However, the CVs of RSCU values among the three tissues based on the top 5% HTGs were still significantly higher than the CVs based on randomly sampled 5% genes, suggesting that codon usage biases of HTGs are truly differential among tissues (Figure 4H).

### tRNA pools adapt better to HTGs in the same tissues but not to tissue-specifically translated genes

**Figure 5:**
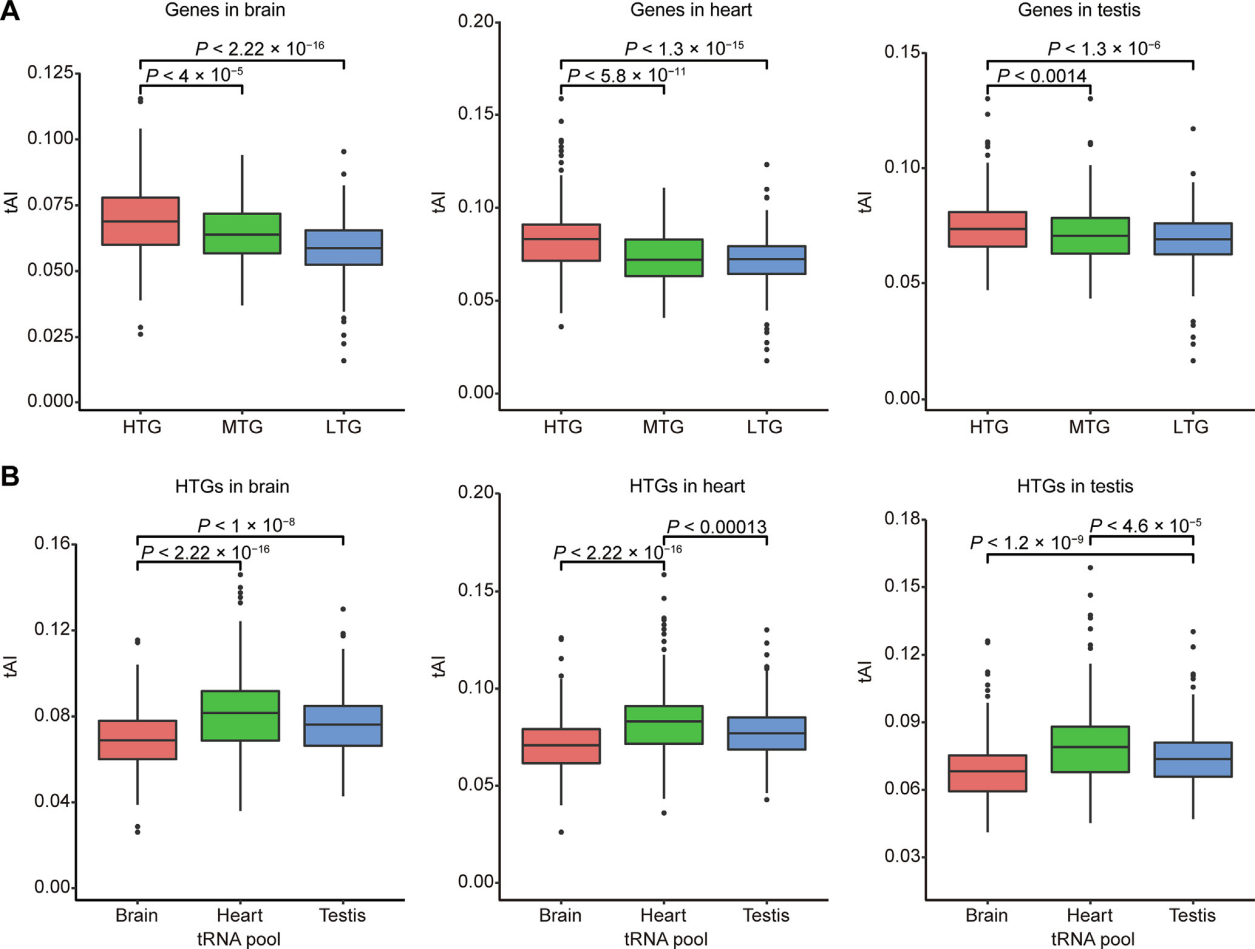
**tRNA pools adapt better to HTGs in the same tissues but not to tissue**-**specifically translated genes** **A.** Box plots comparing the tAI of genes with different TE levels based on the tRNA pools of the same tissues in the three tissues, respectively. **B.** Box plots showing the tAI values of the top 5% HTGs in the brain (left panel), heart (middle panel), and testis (right panel) calculated based on the tRNA pools of the three tissues, respectively. *P* values were calculated by two-tailed Wilcoxon test. tAI, tRNA adaptation index; MTG, moderately translated gene; LTG, lowly translated gene.

**Supplementary Figure S5:**
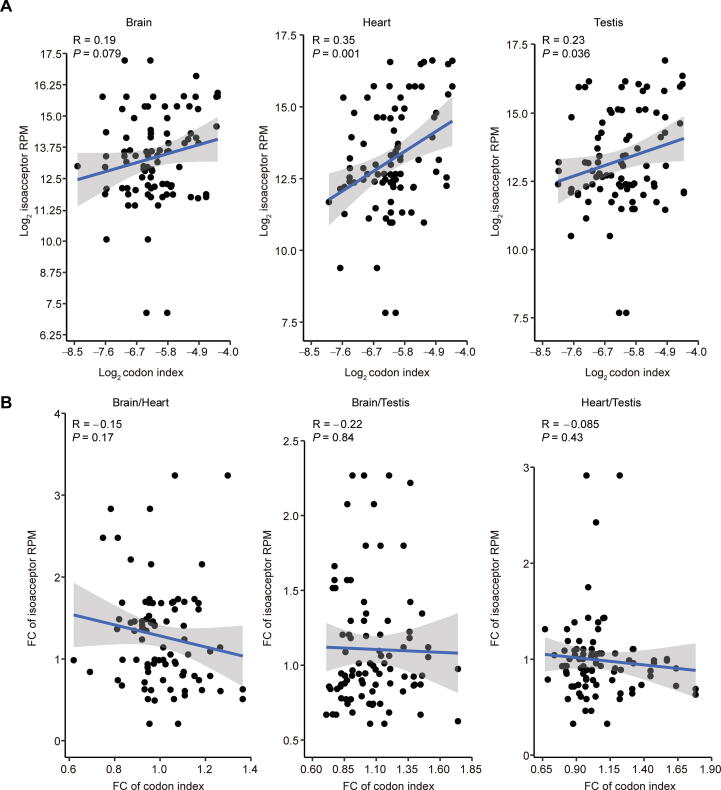
**Pairwise correlation analyses of isoacceptor abundances and the codon compositions among the three tissues A.** Correlation analysis between isoacceptor abundances and the codon compositions. **B.** Correlation analysis between the FCs of isoacceptor abundances and the FC of codon compositions between two tissues.

The more accurate interaction analysis between mRNAs and cognate tRNAs will provide a pivotal way for evaluating effective and accurate translation [33], [34]. To further elucidate the intrinsic relationship between tRNA expression and mRNA translation, we integrated the data of tissue-specific DM-tRNA-seq and RiboTag-seq to comprehensively uncover the correlation between tRNA pools and codon usage bias in HTGs. The adaptation of a specific gene to a specific tRNA pool in terms of codon usage bias can be well evaluated using a widely used metric tAI [35], [36]. We found that the HTGs had significantly higher tAI values than the moderately translated genes (MTGs) and lowly translated genes (LTGs) in all the three tissues based on the tRNA pools of the corresponding tissues (Figure 5A), suggesting the role of tRNAs in regulating translation in the same tissues. However, when we tested the tAI values of HTGs with the tRNA pools from other tissues, we found that the HTGs did not show the highest tAI values based on the tRNA pools of the same tissues. Instead, the tRNA pool of heart had the best adaptation with the HTGs of all tissues (Figure 5B). In addition, we performed the correlation analysis between isoacceptor abundances and the codon compositions of the top 5% HTGs of each tissue based on the general codon–anticodon recognition rules for tRNA genes [36]. Similar to the tAI analyses, we found significant correlations in both the heart and testis but not between tissues (Figure S5A and B). The aforementioned results suggest that although HTGs require more optimal tRNA pools in each tissue, the tissue-specific regulation of tRNA expression is not for the purpose of better adapting the tissue-specific usage biases of synonymous codons. In other words, mammals are not likely to regulate tissue-specific translation of certain genes through regulating the compositions of tRNA pools, which is consistent with the observation that the usage biases of anticodons do not show significant differences among different tissues (Figure 2G).

### tRNA expression correlates with amino acid composition in the same tissues but not between tissues

**Figure 6:**
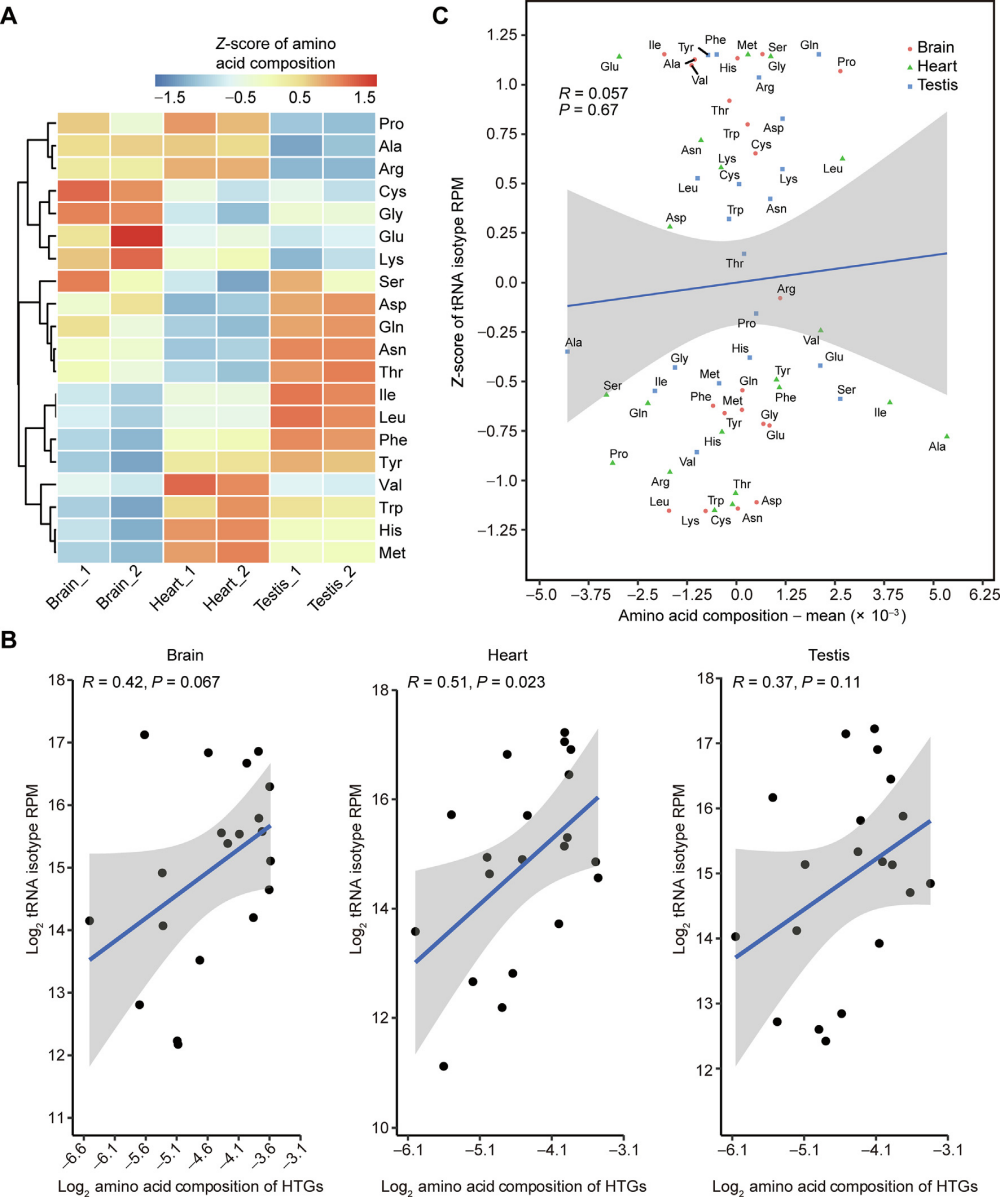
**tRNA expression correlates with amino acid composition in the same tissues but not between tissues** **A.** Heatmap showing the *Z*-scores of amino acid compositions of the top 5% HTGs in the six samples of three mouse tissues. **B.** Scatter plots showing the correlation of amino acid compositions of the top 5% HTGs with the tRNA isotype expression in the brain (left panel), heart (middle panel), and testis (right panel). Blue lines indicate fitted linear models and PCCs are shown. **C.** Scatter plot showing non-significant linear correlation of amino acid compositions subtracted by means with the *Z*-scores of tRNA isotype expression in all three tissues.

**Supplementary Figure S6:**
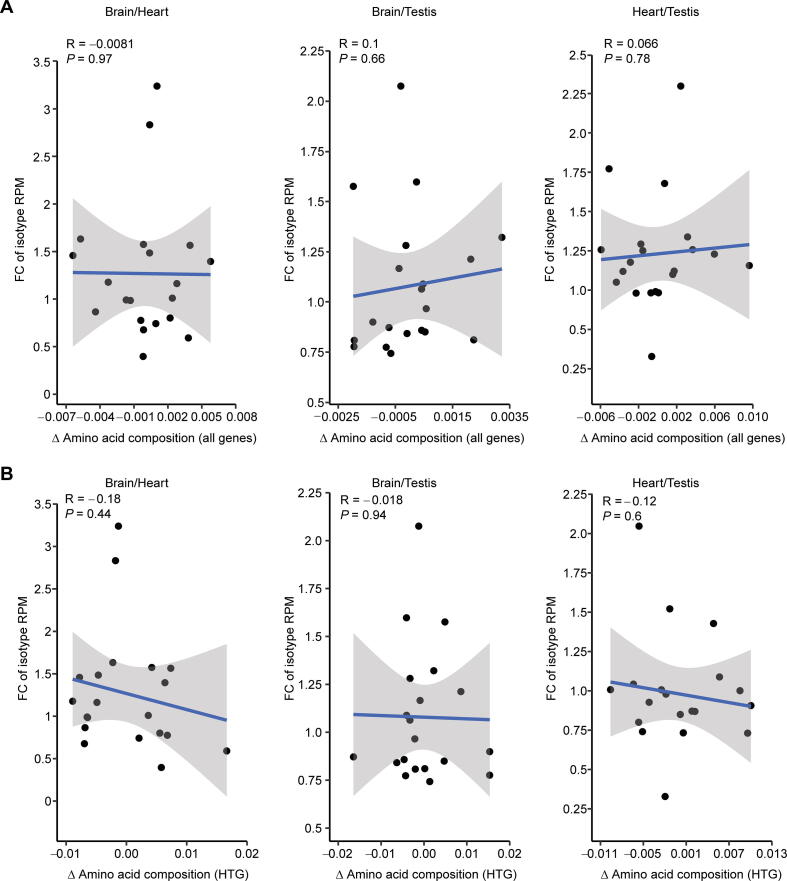
**Pairwise correlation analyses of tRNA isotype expression and amino acid compositions among the three tissues A.** Pairwise correlation analyses of the three tissues between delta amino acid compositions and the FCs of tRNA isotype expression of all genes. **B.** Pairwise correlation analyses of the three tissues between delta amino acid compositions and the FCs of tRNA isotype expression of HTGs.

The analyses of tRNA expression across diverse tissues revealed that isodecoders encoding the same amino acids are likely co-regulated, suggesting that the dynamics of tRNA expression in different tissues might be related with different amino acid compositions of peptides in different tissues. To test this hypothesis, we first tested whether the amino acid compositions are different across the translatomes of different tissues. We calculated each amino acid composition by summing up the number of codons encoding the amino acid of the top 5% HTGs weighted by the translation level (FPKM of IP). As shown in Figure 6A, we observed reproducible tissue-specific amino acid compositions, which is consistent with our observations that the tRNA isotype expression is tissue-specific (Figure 3A). We also found a positive correlation between the amino acid composition and the tRNA isotype expression in heart (*P* = 0.023), and trends of positive correlations in brain (*P* = 0.067) and testis (*P* = 0.11), respectively (Figure 6B). To further address whether the tissue-specific tRNA expression is related to the tissue-specific amino acid compositions of peptides, we tested the correlation of amino acid compositions subtracted by the means with the *Z*-scores of tRNA isotype expression among the three tissues. We observed a non-significant correlation between them (Figure 6C). Non-significant correlations were also observed when we compared the differences of amino acid compositions and the differences of tRNA isotype expression between any two tissues (Figure S6A and B).

We found that the isodecoders encoding the same amino acid were co-regulated across different tissues (Figure 2E). Based on the aforementioned results, this co-regulation is not likely due to active regulatory mechanisms to control the translatomes in a tissue-specific manner. On the contrary, it might be due to post-transcriptional regulation of tRNAs, such as tRNA modifications and aminoacylation, the attachment of amino acids to tRNAs.

## Discussion

Although it is well known that tRNAs play a vital role in the synthesis of protein, whether the tRNA pool correlates well with TE is obscure. Here, based on multiple measurements of tRNAs and translatomes in multiple mouse tissues, we confirmed genuinely dynamic expression of tRNA isodecoder pools as well as isoacceptors among three mouse tissues. Meanwhile, the tRNA pools are significantly correlated with TEs and amino acid compositions of the HTGs in the same tissues but not between tissues. We finally propose that the tissue-specific expression of tRNA may be due to post-transcriptional regulation.

Interestingly, tRNA expression is significantly correlated with TE in the same tissues but not between different tissues. Consistently, several studies have reported that tRNA–codon bias co-adaptation is not tissue-specific but globally driven [13], [37]. These results together suggest the organisms may not regulate the translation of specific genes tissue-specifically through regulating tRNA expression, probably due to the difficulty of achieving precise adjustment through the regulation of tRNA expression. Nevertheless, we cannot rule out that there may be a weak correlation to be revealed and more accurate detection methods need to be developed in the future.

It has advantages of using RiboTag-seq to measure the TEs in this study. In contrast to ribosome profiling (Ribo-seq), which measures the translation through obtaining the mRNA fragments protected by ribosomes [38], the RiboTag-seq takes advantage of RPL22, a component of the 60S subunit of the ribosome, to pull down the mRNAs involved in translation elongation. In principle, Ribo-seq has difficulty in distinguishing the large and small ribosome subunits, and thus cannot distinguish translation initiation and elongation. In contrast, RiboTag-seq captures full-length mRNAs bound by actively translating polysomes, thus providing a more specific measurement of translation elongation. Since translation initiation and elongation may relate to TE in different manners [39], RiboTag-seq overcomes the drawback of Ribo-seq. In addition, considering that we have found a significant correlation between tAI and TE, the RiboTag-seq technology used in this study is reliable in representing the translatome [28].

In this study, we hypothesize that the difference of tRNA between tissues is due to passive post-transcriptional regulation during the process of tRNA maturation. First, we found that the isodecoders encoding the same amino acid are co-regulated. Second, there is no difference of RNAPIII binding on tRNA genes at the isoacceptor level among tissues [7], suggesting that differences in tRNA may be related to post-transcriptional regulation. In addition, it was reported that in *Escherichia coli*, tRNA can be destabilized and degraded in the case of amino acid starvation and upon the demand for protein synthesis decreases, suggesting that the content of tRNA is related to the concentration of the free amino acids [26]. Meanwhile, several groups have found that certain amino acids such as cysteine [40], glycine [41], serine [42], and threonyl [43] have key impacts on the modifications of tRNAs, and some modifications of tRNA will further affect tRNA abundances [44], [45]. Therefore, post-transcriptional regulation of tRNAs may also contribute to the tissue-specific expression of tRNAs and translatomes. This manner of tRNA regulation passively fine-tunes the tRNA expression in a tissue-specific manner but not for the purpose of regulating the translatomes.

One possible post-transcriptional regulation that may result in tRNA differences between tissues is through the aminoacylation process, which might be regulated by free amino acid concentrations and the activities of aminoacyl-tRNA synthetases (aaRSs). The activities of aaRSs are dynamic [46]. Mammals have 20 cytosolic aaRSs, which are the enzymes that attach amino acids to tRNAs and thus allow tRNA molecules to act as adaptors to decode mRNAs. Individual tRNA isotype is aminoacylated by a specific aaRS. The aminoacylated tRNA is captured by a translation elongation factor and it is delivered to the ribosome for protein synthesis. The expression of tRNA isotypes and free amino acid concentrations may affect the levels of aminoacyl-tRNAs, which in turn may have positive or negative feedback on the early processing steps of tRNAs or affect the stability of tRNAs in a tissue-specific manner, thus leading to the observed dynamic expression of tRNAs.

Another post-transcriptional regulation that may result in tRNA differences between tissues is through tRNA modification. tRNAs are the most generally modified RNA species in cells. Eukaryotic tRNAs contain an average of 13 modified bases per molecule. Modifications occurring in the anticodon loop are essential to regulate mRNA decoding, while modifications outside of the anticodon loop are vital to regulate tRNA stability, tRNA localization, and tRNA folding [23]. Dynamic variations at the levels of tRNA modifications play important roles in regulating the TEs and translation accuracies of particular genes that rely on the codon usages. However, the profiling of tissue-specific tRNA modification is still lacking. In the future, the development of novel large-scale methods to reveal the tRNA modification level can point the light way to understand the diverse function of tRNAs during translation process.

**Supplementary Figure S7:**
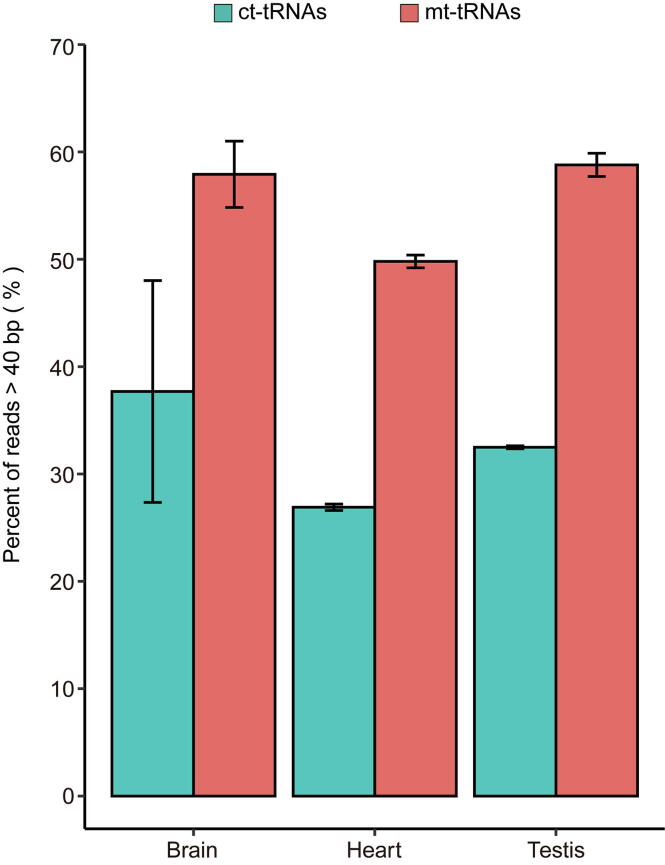
**Comparison of the percentages of reads with lengths greater than 40 bp between ct-tRNAs and mt-tRNAs**

Since it is known that DM-tRNA-seq can also generate a large fraction of incomplete tRNA reads due to the incomplete erasure of the modifications on tRNAs [22], the difference of tRNA read length also reflects the differences of modifications. According to the percent of reads with length > 40 bp, we found the proportions are quite similar between different tissues but the proportion of mt-tRNAs is larger than ct-tRNAs (Figure S7). This result is consistent with the previous report that ct-tRNAs and mt-tRNAs are modified differently. mt-tRNAs of higher eukaryotes have smaller and shorter stem and loop regions than those of ct-tRNAs [23]. Modifications in mt-tRNAs are less diverse comparing with ct-tRNAs [41], [47]. m^1^A9 and m^2^G10 are considerably abundant modifications identified in mt-tRNA species [41], which can be removed by AlkB demethylases [27] and result in longer mt-tRNAs reads in DM-tRNA-seq.

**Supplementary Figure S8:**
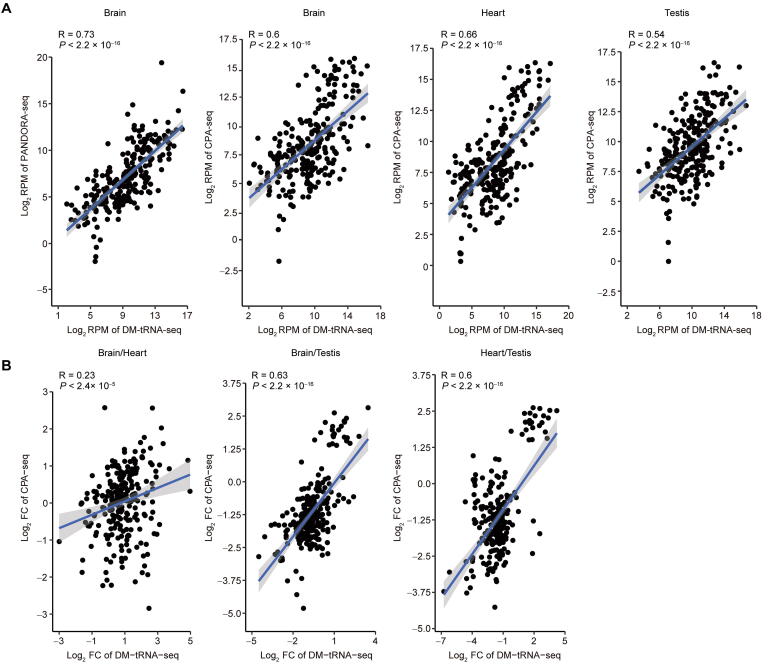
**Correlation analyses of the expression of tRNAs and tsRNAs among the three tissues A.** Correlation analysis of the isodecoder RPMs between tsRNA and tRNA. **B.** Correlation analysis between the FCs of tsRNA and the FCs of tRNA.

In addition, tRNA modifications also contribute to different biogenesis of tRNA-derived small RNAs (tsRNAs), which are known to regulate translation in versatile ways [48]. Based on the expression of tsRNAs in the brain, heart, and testis examined by the PANDORA-seq [49] and CPA-seq [50], we found the expression of tsRNAs was significantly and positively correlated with the expression of tRNAs in the same tissues and between different tissues (Figure S8A and B). The results suggest that tissue-specific expression of tRNA might be related to tsRNAs. It is possible that there might be unknown mechanisms that dynamically regulate the expression of tRNAs in different tissues in order to dynamically generate tsRNA in different tissues.

## Materials and methods

### Animals

Mice were maintained on a 12-h light/12-h dark cycle. The *RiboTag* mice (Stock No. 011029, Jackson Laboratory, Bar Harbor, ME) and *CMV-Cre* mice (Stock No. 006054, Jackson Laboratory, Bar Harbor, ME) were purchased from Jackson Laboratory. The *RiboTag* mice were bred to the *CMV-Cre* mice to obtain homozygous mice constitutively expressing *Rpl22-HA*. Once the model of *Rpl22-HA*-expressing homozygous mice was built successfully, we maintained the colony as a separate mouse line.

### Tissue sample preparation and RNA isolation

All mouse tissue samples were isolated from adult male *CMV-Cre:RiboTag* mice using procedures approved by the Animal Research Committee of the First Affiliated Hospital, Sun Yat-sen University, China. Samples were rapidly frozen in liquid nitrogen and stored at −80 °C until use. Then, 1 ml of TRIzol (Catalog No. 15596026, Invitrogen, Carlsbad, CA) was added per 100 mg of dissected whole tissue, and samples were homogenized in TRIzol buffer with a homogenizer (Catalog No. 2010, Jingxin, Shanghai, China) until the suspension was completely homogeneous. Cell debris was removed by a high-speed centrifugation procedure. RNA was isolated according to the manufacturer’s instructions of TRIzol reagent, resuspended in nuclease-free water, and stored at −80 °C until DM-tRNA-seq.

### Recombinant protein purification

Recombinant wild-type and D135S AlkB proteins were purified as previously described [51]. pET30a-AlkB and pET30a-AlkB-D135S were transformed into BL21 bacteria for induced expression of recombinant proteins. Bacteria were inoculated and cultured on lysogeny brothmedium (LB; Catalog No. ST156, Beyotime Biotechnology, Shanghai, China) at 37 °C. The expression of recombinant wild-type and D135S AlkB proteins was induced in BL21 bacteria (OD_600_ = 0.6–0.7) using 0.5 mM isopropyl β-D-thiogalactoside (IPTG; Catalog No. I5502, Sigma, St. Louis, MO) at 20 °C overnight. Then the bacteria were collected and lysed by sonication, centrifuged at 15,000 r/min at 4 °C for 60 min. The supernatant was collected for the purification of recombinant proteins using Ni-NTA Agarose (Catalog No. 30210, Qiagen, Alameda, CA) following the manufacturer’s instructions and stored at −80 °C.

### DM-tRNA-seq

DM-tRNA-seq was performed following the previously reported protocol [27], [47] with some modifications. Small RNAs (< 200 nt) were first purified using the Quick-RNA Microprep kit (Catalog No. R1050, Zymo Research, Orange, CA). Isolated small RNAs were treated with recombinant wild-type and D135S AlkB proteins to remove the dominant methylations on RNAs. Then, demethylated RNAs were purified with Oligo Clean & Concentrator kit (Catalog No. D4060, Zymo Research). After that, AlkB-treated RNA libraries were constructed with NEBNext Small RNA Library Prep Set (Catalog No. E7330S, New England Biolabs, Ipswich, MA). The cDNA libraries were sequenced on Illumina HiSeq X10 with paired-end 2 × 150 bp read length.

### Western blot

Tissue-specific lysates were extracted with radioimmunoprecipitation assay (RIPA) buffer by a homogenizer. Western blot assays were performed as described previously [52]. Nitrocellulose membranes were blocked using 5% Blotting Grade Blocker Non-Fat Dry Milk (Catalog No. 1706404XTU, Bio-Rad, Hercules, CA) and were then incubated with primary antibody at 4 °C overnight. For primary antibodies used were as follows: anti-HA tag (Catalog No. ab9110, Abcam, Cambridge, UK), anti-IgG (Catalog No. B900620, Proteintech, Wuhan, China), and anti-tubulin (Catalog No. 11224-1-AP, Proteintech). The blots were then incubated with horseradish peroxidase-conjugated secondary antibody (Catalog No. 7074, Cell Signaling Technology, Berkeley, CA) at room temperature for 1 h, and the proteins were then detected using the electrogenerated chemiluminescence (ECL) chemiluminescence system (Catalog No. 4600, Tanon, Shanghai, China).

### Polysome immunoprecipitation

RiboTag immunoprecipitation was performed as previously described [31] with some modifications. Tissue samples were extracted from *CMV-Cre:RiboTag* mice, flash-frozen in liquid nitrogen, and stored at −80 °C until use. Tissues were homogenized in ice-cold homogenization buffer [50 mM Tris-HCl pH 7.4, 1% NP-40, 100 mM KCl, 12 mM MgCl_2_, 100 μg/ml cycloheximide (Catalog No. 66819, Sigma), 1:100 protease inhibitor cocktail (Catalog No. 4693116001, Roche, Mannheim, Germany), 1 mg/ml Heparin, 1 mM dithiothreitol (DTT), 200 U/ml RNasin (Catalog No. N2111, Promega, Madison, WI) in RNase-free water] with a homogenizer until the suspension was completely homogeneous. To remove cell debris, the homogenate was transferred to a microcentrifuge tube and centrifuged at 13,000 *g* at 4 °C for 15 min. Supernatants were transferred to a fresh microcentrifuge tube on ice, and then 70 μl was removed for input fraction analysis and 8 μl (8 µg) of anti-HA antibody was added to the supernatant, followed by 4 h of incubation with slow rotation in a cold room at 4 °C. Meanwhile, Pierce Protein A/G Magnetic Beads (Catalog No. 88803, ThermoFisher Scientific, Waltham, MA), 80 μl per sample, were equilibrated to homogenization buffer by washing three times. At the end of 4 h of incubation with antibody, beads were added to each sample, followed by incubation overnight at 4 °C. The following day, samples were placed in a magnet on ice, and supernatants were recovered before washing the pellets three times for 10 min in high salt buffer (50 mM Tris-HCl pH 7.4, 1% NP-40, 300 mM KCl, 12 mM MgCl_2_, 100 μg/ml cycloheximide, 1 mM DTT). At the end of the washes, beads were magnetized and excess buffer was removed. To prepare total RNA, 5 volumes of Qiagen RLT buffer (Catalog No. 79216, Qiagen) were added to the remaining pellets or the input samples. Total RNA was prepared according to the manufacturer’s instructions using RNeasy Mini Kit (Catalog No. 74104, Qiagen), quantified with a NanoDrop 2000 spectrophotometer (Catalog No. ND2000USCAN, ThermoFisher Scientific), and taken for RNA sequencing (RNA-seq). For high-throughput sequencing, both input and IP samples were used for library construction with the SMARTer Stranded Total RNA-seq Kit v2 (Catalog No. 635005, Takara, Dalian, China), and single-end 50-base reads were generated on the BGISEQ500 platform.

### Processing of high-throughput sequencing data

The nuclear tRNA and mt-tRNA reference sequences were downloaded from GtRNAdb [4] and mt-RNA database mitotRNAdb [29], respectively. Nuclear tRNAs and mt-tRNAs with unique sequences generated by collapsing the identical tRNAs were merged and used as the reference for downstream mapping. DM-tRNA-seq raw reads were first processed using Cutadapt (v1.18) to remove adaptor sequences and 3′-CCA sequences, and to discard reads shorter than 25 nt. Then, Bowtie2 (v2.3.5) [53] was used to align the adaptor-trimmed and filtered reads to the tRNA reference sequences of the mouse genome (mm10) with the parameters: --min-score G,1,8 --local -D 20 -R 3 -N 1 -L 10 -I S,1,0.5. Only reads with unique hits and mapping quality > 10 were considered for further analysis. The RPMs of isodecoders were calculated by multiplying the number of reads mapped to the gene by 1 × 10^6^ and dividing it by the total number of mapped reads. The anticodon-level or amino acid-level counts were calculated by summing up the counts of isodecoders with the same anticodons or encoding the same amino acids. tRNA-seq read count tables at both the anticodon level and isodecoder level were used to perform differential tRNA expression analysis between each two of the three mouse tissues using the DESeq2 [30]. Differentially expressed tRNAs were determined by requiring FDR < 0.05 between any two tissues. The same pipeline was also applied to the public data of PANDORA-seq [49] and CPA-seq [50] to calculate the total RPM of tsRNA derived from each tRNA isodecoder.

RiboTag-seq raw reads were first mapped to rRNA reference sequences using Bowtie2 (v2.3.5). Reads that were mapped to rRNAs were discarded. The remaining reads were then mapped to the mouse genome (mm10) using STAR (v2.7.5). Only uniquely mapped reads were considered for further analysis. Gene expression was calculated using StringTie v1.3.5.

### Metric definition

Codon index was defined to measure the usage of the *i-*th codon and calculated as follows:

(1) $$C\mathrm{odon}\;index_{i}\;=\;\frac{\sum_{j\;=\;1\;}^{m}\frac{x_{\mathrm{ij}}}{\sum_{i\;\text{= 1}}^{n}\;x_{\mathrm{ij}}}\;\times \;IP\;FPKM_{j}}{\sum_{i\;=\;1}^{n}\sum_{j\;=\;1}^{m}\frac{x_{\mathrm{ij}}}{\sum_{i\;=1}^{n}\;x_{\mathrm{ij}}}\;\times \;\mathrm{IP}\;FPKM_{j}}$$

Here, *x_ij_* denotes the number of occurrences of the *i-*th codon in the *j-*th gene, *IP FPKM_j_* denotes the FPKM value of the *j-*th gene in IP of RiboTag-seq, *n* denotes the number of codons, and *m* denotes the number of genes.

RSCU was defined by modifying the previously defined RSCU by Sharp et al. [32] with IP FPKM as the weight of each gene. It was calculated for the *j-*th codon for the *i-*th amino acid as follows:

(2) $${\mathrm{RSCU}}_{\mathrm{ij}}\;=\;\frac{n_{i}\;\times \sum_{k\;=\;1\;}^{m}x_{\mathrm{ijk}}\times {\;IP\;FPKM}_{k}}{\sum_{j\;=\;1}^{n_{i}}\sum_{k=\;1\;}^{m}x_{\mathrm{ijk}}\times {\;IP\;FPKM}_{k}}$$

Here, *n_i_* denotes the number of the synonymous codon for the *i-*th amino acid, *x_ijk_* denotes the number of occurrences of the *j-*th codon for the *i-*th amino acid in the *k-*th gene, *m* denotes the number of genes, and *IP FPKM_k_* denotes the FPKM value of the *k-*th gene in IP of RiboTag-seq.

RSAU was calculated for the *j-*th anticodon for the *i-*th amino acid as follows:

(3) $${\mathrm{RSAU}}_{\mathrm{ij}}\;=\;\frac{n_{i}\;\times \;x_{\mathrm{ij}}}{\sum_{j\;=\;1}^{n_{i}}x_{\mathrm{ij}}}$$

Here, *n_i_* denotes the number of the anticodon for the *i-*th amino acid, and *x_ij_* denotes the RPM of the *j-*th anticodon for the *i-*th amino acid.

RSIU was calculated for the *j-*th isodecoder for the *i-*th anticodon as follows:

(4) $${\mathrm{RSIU}}_{\mathrm{ij}}\;=\;\frac{n_{i}\;\times \;x_{\mathrm{ij}}}{\sum_{j\;=\;1}^{n_{i}}x_{\mathrm{ij}}}$$

Here, *n_i_* denotes the number of the isodecoders for the *i*-th anticodon, and *x_ij_* denotes the RPM of the *j*-th isodecoder for the *i-*th anticodon.

Amino acid composition was calculated for the *i-*th amino acid as follows:

(5) $$A\mathrm{mino}\;acid\;composition_{i}\;=\;\overset{m}{\underset{j=1}{\sum }}\mathrm{IP}\;FPKM_{j}\;\times \;n_{i}$$

Here, *n_i_* denotes the number of codons encoding the *i-*th amino acid for the *j-*th gene, *IP FPKM_j_* denotes the FPKM value of the *j-*th gene in IP of RiboTag-seq, and *m* denotes the number of genes used in the calculation.

### tRNA and translatome analyses

Permutation was performed by randomly switching the anticodons of the isodecoders and regrouping them into anticodons according to the permutated anticodons. We compared the mean CVs of anticodon expression, RSAU values, and tRNA isotype expression with 10,000 times of permutations. The *P* values of permutation analyses were determined by calculating the fraction of the RSAU values of permutations greater (isoacceptor expression, isotype expression) or less (RSAU) than the observed data.

TEs were calculated as the ratios between the FPKMs of IPs and the inputs of RiboTag-seq. Only the genes with FPKM > 1 in both input and IP samples were used in the downstream analyses. RSCU values were calculated as previously described by Sharp et al*.* [32] based on the top 5% HTGs. The coding region of the longest coding isoform of each gene was used for codon analyses. For comparison, RSCU values based on randomly sampled 5% genes with FPKM > 1 in both input and IP samples were also calculated. Significance was determined by Wilcoxon signed-rank test.

tAI was calculated by R package tAI [36]. tAIs using different tRNA pools were calculated for genes with the top 5%, medium 5%, and bottom 5% of TEs. The significance between them was based on Wilcoxon signed-rank test. Data visualization and plotting were performed using ggplot2, ggrepel, and ggforce R packages.

The correlation analyses between isoacceptor abundances and the codon compositions of the top 5% HTGs of each tissue were based on the general codon–anticodon recognition rules for tRNA genes [36]. Codons recognized by multiple anticodons as well as anticodons that recognize multiple codons were repeated to form one-to-one codon–anticodon pairs.

## Ethical statement

Animal experiments were licensed with the Approval No. SYSU-IACUC-2021-000089 and performed in agreement with the guidelines of the Animal Research Committee of the First Affiliated Hospital, Sun Yat-sen University, China.

## Code availability

Source codes used for processing and analyzing the DM-tRNA-seq and RiboTag-seq data have been submitted to BioCode at the National Genomics Data Center (NGDC), Beijing Institute of Genomics (BIG), Chinese Academy of Sciences (CAS) / China National Center for Bioinformation (CNCB) (BioCode: BT007304), and are publicly accessible at https://ngdc.cncb.ac.cn/biocode/tools/BT007304.

## Data availability

The raw sequencing data of DM-tRNA-seq and RiboTag-seq in this study have been deposited in the Genome Sequence Archive [54] at the NGDC, BIG, CAS / CNCB (GSA: CRA005907), and are publicly accessible at https://ngdc.cncb.ac.cn/gsa.

## Competing interests

The authors have declared no competing interests.

## CRediT authorship contribution statement

**Peng Yu:** Methodology, Validation, Visualization, Writing – original draft. **Siting Zhou:** Software, Formal analysis, Visualization, Writing – original draft. **Yan Gao:** Investigation, Funding acquisition. **Yu Liang:** Investigation. **Wenbing Guo:** Formal analysis. **Dan Ohtan Wang:** Writing – review & editing. **Shuaiwen Ding:** Writing – review & editing. **Shuibin Lin:** Conceptualization, Writing – review & editing, Supervision, Project administration, Funding acquisition. **Jinkai Wang:** Conceptualization, Writing – review & editing, Supervision, Project administration, Funding acquisition. **Yixian Cun:** Conceptualization, Writing – review & editing, Supervision, Project administration. All authors have read and approved the final manuscript.

## Supplementary material

Supplementary Table S1 **The counts and expression of tRNAs**

Supplementary Table S2 **The gene FPKMs of IP and input of RiboTag-seq**
